# Optimization and Characterization of Silver Nanoparticle by Endophytic Fungi *Penicillium* sp. Isolated from *Curcuma longa* (Turmeric) and Application Studies against MDR *E. coli* and *S. aureus*

**DOI:** 10.1155/2014/408021

**Published:** 2014-02-03

**Authors:** Dattu Singh, Vandana Rathod, Shivaraj Ninganagouda, Jyothi Hiremath, Ashish Kumar Singh, Jasmine Mathew

**Affiliations:** Department of Microbiology, Gulbarga University, Gulbarga, Karnataka 585106, India

## Abstract

Development of ecofriendly and reliable processes for the synthesis of nanoparticles has attracted considerable interest in nanotechnology because of its tremendous impetus in modulating metals into nanosize to their potential use for human benefits. In this study an endophytic fungus, *Penicillium* sp., isolated from healthy leaves of *Curcuma longa* (turmeric) was subjected to extracellular biosynthesis of silver nanoparticles (AgNps) and their activity against MDR *E. coli* and *S. aureus*. The biosynthesized AgNps optimization was studied and characterized by UV-visible spectroscopy, Fourier transform infrared spectroscopy (FTIR), and transmission electron microscopy (TEM). Then produced AgNps were tested against MDR *E. coli* and *S. aureus*. The endophytic fungus *Penicillium* sp. from healthy leaves of *C. longa* (turmeric) was found to be a good producer of AgNps. Parametric optimization showed maximum absorbance of 420–425 nm at pH-7, 25°C with 1 mM AgNO_3_ concentration and 15–20 g of wet biomass. Further TEM revealed the formation of spherical, well-dispersed nanoparticles with size ranging between 25 and 30 nm and FTIR shows the bands at 1644 and 1538 cm^−1^ corresponding to the binding vibrations of amide I and II bands of proteins, respectively. Antibacterial activity against MDR *E. coli* and *S. aureus* showed good results showing maximum zone of inhibition of 17 mm and 16 mm, respectively, at 80 *µ*L of AgNps.

## 1. Introduction

Resistance in human pathogens is a big challenge in fields like pharmaceutical and biomedicine. Antibiotic resistance profiles lead to fear about the emergence and reemergence of multidrug-resistant (MDR) pathogens [1]. These MDR pathogens require multiple treatments of broad-spectrum antibiotics, which are less effective, more toxic, and more expensive [2]. Therefore, development of or modification in antimicrobial compounds to improve bactericidal potential is a priority area of research in the modern era [3]. Nanotechnology is an emerging field with its application in science and technology for the purpose of synthesis and development of nanomaterials at the nanoscale level [4]. The use of metal nanoparticles is gaining impetus in the present century due to their optical, electrical, and catalytic properties. To utilize and optimize physical properties of nanosized metal particles large spectrums of research have been focused to control the size and shape, which is crucial in tuning their physical, chemical, and optical properties [5–7].

Various techniques, such as chemical, physical, and mechanical techniques, have been developed to prepare metal nanoparticles, as these methods are costly, toxic, and nonecofriendly. A green synthesis of nanoparticles with the help of biological sources like plant and microorganisms is carried out because they are less toxic to human and environment [8]. Different types of metal nanoparticles are produced, copper, zinc, titanium [9], magnesium, gold [10], alginate [11], and silver; of them silver nanoparticles (AgNps) have proved to be most effective as they have good antimicrobial efficacy against bacteria, viruses, and other eukaryotic microorganisms [12]. The researchers are moving towards nanoparticles especially silver nanoparticles to solve the problem of emerging human pathogens [13, 14]. Silver nanoparticles are more effective because of the high surface area to volume ratio so that a large proportion of silver nanoparticles are in direct contact with their environment [15]. By using fungi and bacteria various studies on biosynthesis of silver nanoparticles have been demonstrated [16, 17]; one such clique of microbes is the endophytes whose potential biosynthesis of nanoparticles has not been studied completely. Bacon et al. [18] defined endophytes as “microbes that colonize living internal tissues of plants without causing any immediate, overt negative effects,” whereas Strobel and colleagues [19] suggested that the relationship can range from mutualistic to bordering on pathogens. Very few reports are available wherein endophytic fungi were used for the synthesis of nanoparticles. Therefore, in the present study, we have used endophytic fungi *Penicillium *sp. isolated from healthy leaves of *Curcuma longa* (turmeric) for the extracellular biosynthesis of silver nanoparticles, its optimization, and characterization studies; to obtain monodispersed silver nanoparticles its efficacy against MDR *E. coli* and *S. aureus *strains, was studied.

## 2. Materials and Methods

### 2.1. Isolation of Endophytic Fungi

Healthy leaves of *Curcuma longa* (turmeric) were collected from the Department of Botany, Gulbarga University, Gulbarga. The leaves brought to the laboratory were washed several times under running tap water and cut into small pieces. These pieces were surface sterilized by sequential rinsing into 70% ethanol (C_2_H_5_OH) for 30 sec, 0.01% mercuric chloride (HgCl_2_) for 5 min, 0.5% sodium hypochlorite (NaOCl), and 2-3 minutes with sterile distilled water and then allowed to dry under sterile conditions. The cut surface of the segment was placed on petri dish containing (potato dextrose agar) PDA supplemented with streptomycin sulfate (250 *μ*g/mL), incubated at 28°C for 3-4 days, and monitored every day for the growth of endophytic fungal colony from leaf segment. The fungi which grew out from leaf segment were isolated and brought into pure culture onto other PDA plates. The fungal isolate was identified based on its morphological and reproductive characters using standard identification manual [20].

### 2.2. Extracellular Synthesis of Silver Nanoparticles

The isolated endophytic fungus, *Penicillium *sp., was grown in 250 mL Erlenmeyer flask. About 100 mL of malt glucose yeast peptone (MGYP) broth [21] contained yeast extract and malt extract, 0.3% each, glucose, 1%, and peptone, 0.5%, at 25°C in static position. After 72 h of incubation the mycelial biomass was separated by filtration and then extensively washed with distilled water to remove the traces of media components. This biomass was taken into flasks containing 100 mL distilled water and incubated at the same position for 48 h. The suspension was filtered with Whatman filter paper number 1 and was used. Further, the fungal filtrate was mixed with aqueous solution AgNO_3_ of 1 mM concentration for reduction [20].

### 2.3. Optimization Studies for Silver Nanoparticles Production

There is always a continuous interaction between organism and the environment in which they live. The environmental conditions exert an influence on growth and development of organism. The enzyme production by fungi is influenced by the condition in which the organisms are cultivated [21, 22]. Therefore, optimization studies will not only support good growth but also enhance product yield.

#### 2.3.1. Effect of AgNO_3_ Concentration

The production of nanoparticles is also dependent on substrate concentration. The concentration of AgNO_3_ from 0.5 to 2.0 mM was studied with a difference of 0.5 mM. The optimum concentration for the synthesis of nanosilver is confirmed by UV-visible absorption spectroscopy.

#### 2.3.2. Effect of pH

pH has a strong influence on growth and enzyme production which is required for the biosynthesis of AgNps. Different pH ranging from 4.0 to 8.0 was used with the difference of 1.0 to study the influence of pH on AgNps production from endophytic fungus, *Penicillium *sp.

#### 2.3.3. Effect of Temperature

Temperature plays a very important role in all reactions. Optimization studies with respect to temperature were carried out with temperature ranging from 20°C to 40°C with difference of 5°C on endophytic fungus, *Penicillium *sp. for AgNps production. The sample was analyzed with UV-visible absorption spectroscopy and further effect of temperature on nanoparticles was studied.

#### 2.3.4. Effect of Biomass Concentration

The effect of biomass concentration on the extracellular synthesis of AgNps was studied by exposing 5 to 20 g of wet biomass with a difference of 5 g of endophytic fungi *Penicillium *sp. Biosynthesis of nanosilver particles at different biomass concentrations was characterized by UV-visible absorption spectroscopy.

### 2.4. Characterization Studies for Silver Nanoparticles

#### 2.4.1. UV-Visible Spectroscopy

The formation of AgNps was preliminarily confirmed by visual observation of color change from pale white to reddish brown, further by UV-visible spectra at different time intervals. Sharp peak given by UV-visible spectrum confirms silver nanoparticle at the absorption range between 400 and 450 nm.

#### 2.4.2. Transmission Electron Microscopy (TEM)

Characterization of AgNps was done by TEM (Hitachi-H-7500) to know the size and shape of nanoparticles. The samples were prepared by drop-coating the AgNps solution onto the carbon-coated copper grid and were loaded onto a specimen holder. TEM micrographs were taken and then sizes and shape of AgNps were confirmed.

#### 2.4.3. Fourier Transform Infrared Spectroscopy (FTIR)

The AgNps synthesized were air-dried at room temperature and were subjected to FTIR analysis in the range of 500 to 4000 cm^−1^. The probable biomolecules responsible for reduction, capping, and effective stabilization of the AgNps were recorded using FTIR spectrophotometer at diffuse reflectance mode.

### 2.5. Analysis of Antibacterial Activity of Silver Nanoparticles against MDR *E. coli* and *S. aureus* Strains

#### 2.5.1. Antimicrobial Susceptibility Assay of Clinical Isolates

The clinical isolates were collected from the diagnostic labs of the Gulbarga district and tested for the sensitivity against different antibiotics by subculturing the bacterial cultures, incubated at 37°C for 5-6 h to moderate turbidity. Then lawn of pathogenic bacteria was prepared on nutrient agar plate using sterile swabs and then antibiotic disc was kept aseptically on the lawn plate and incubated at 37°C for 24 h and then results were monitored.

#### 2.5.2. Antibacterial Assay of Silver Nanoparticles against MDR *E. coli* and *S. aureus*

Antibacterial assay was done on MDR *E. coli* and *S. aureus* strains using agar well diffusion assay method [23, 24]. The test organisms were grown in nutrient broth for 5-6 h lawn of MDR bacteria which was prepared on nutrient agar plates using sterile swabs. Agar wells were made on nutrient agar plates using gel puncture and each well was loaded with 20 *μ*L, 40 *μ*L, 60 *μ*L, and 80 *μ*L, respectively, of AgNps solution and incubated at 37°C for 24 h. The zone of inhibition was measured.

## 3. Results and Discussion

### 3.1. Isolation of Endophytic Fungi

**Figure 1:**
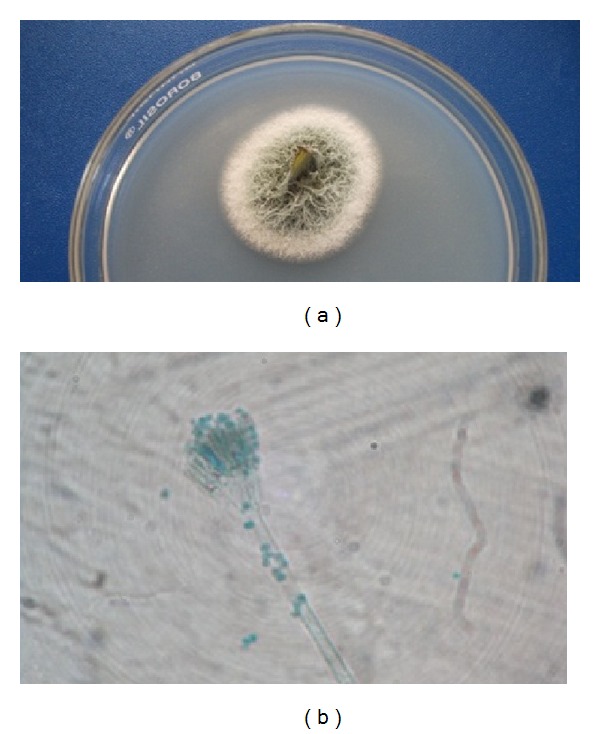
(a) Endophytic fungi grown from sterilized leaf segment of *Curcuma longa* on PDA. (b) Microscopic image of endophytic fungi, *Penicillium *sp.

The surface sterilized leaf segments of *C. longa* (turmeric) were placed on PDA medium and incubated for 72 h. The fungi grown out from tissue were brought into pure culture, identified by microscopic observation and morphological characteristic features. Studies revealed that the fungus is *Penicillium *sp.  Figure 1.

### 3.2. Extracellular Synthesis of Silver Nanoparticles

**Figure 2:**
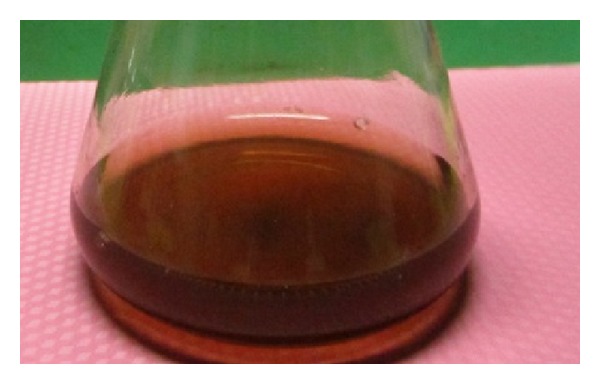
Color change to reddish brown after treating filtrate of endophytic fungi, *Penicillium* sp., with 1 mM AgNO_3_.

Fungal filtrate was treated with equal volume of  1 mM AgNO_3_ solution after 24 h incubation; appearance of color change from pale white to brown is a clear indication for the formation of silver nanoparticles in the reaction mixture. The intensity of the color was increased with the period of incubation Figure 2. The appearance of the brown color was due to the excitation of surface plasmon vibrations [25].

### 3.3. Optimization Studies for Silver Nanoparticles Production

Environmental conditions profoundly modulate the growth and metabolism of fungi. Culture conditions have been the critical components, directly affecting the productivity and also the process economics. Optimization of physical parameters will not only support good growth but also enhance the product yield [26]. The growth conditions, such as substrate concentration, pH, temperature, and inoculum size, directly monitor the rate of enzyme activity which reflects the synthesis of AgNps.

#### 3.3.1. Effect of AgNO_3_ Concentration

**Figure 3:**
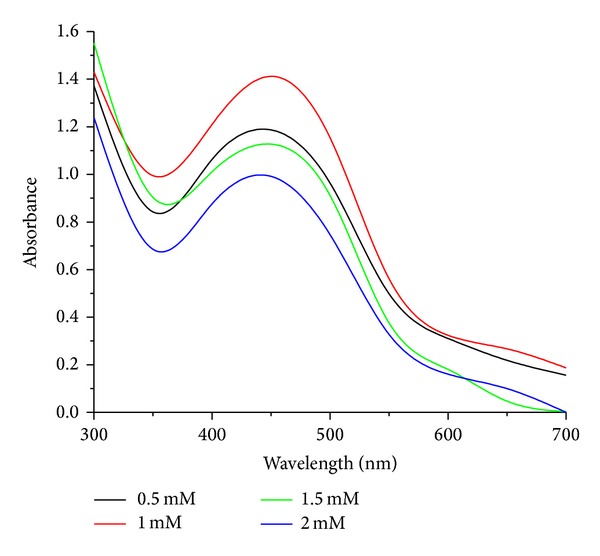
UV-visible absorption spectra of AgNps at different concentrations of AgNO_3_.

Biosynthesis of AgNps with different concentrations of silver nitrate solution 0.5 mM to 2 mM was studied with the fungal filtrate. The optimum substrate concentration was predicted as 1 mM by color change and by UV-visible spectra at the maximum absorbance peak of 425 nm Figure 3. When the AgNO_3_ concentration increased to 2 mM the particle size may increase due to the aggregation of large silver nanoparticles [27]. Our results correlate with Banu et al., [25] who used 1.0 mM AgNO_3_ for the production of AgNps using the fungus *Rhizopus stolonifer*.

#### 3.3.2. Effect of pH

**Figure 4:**
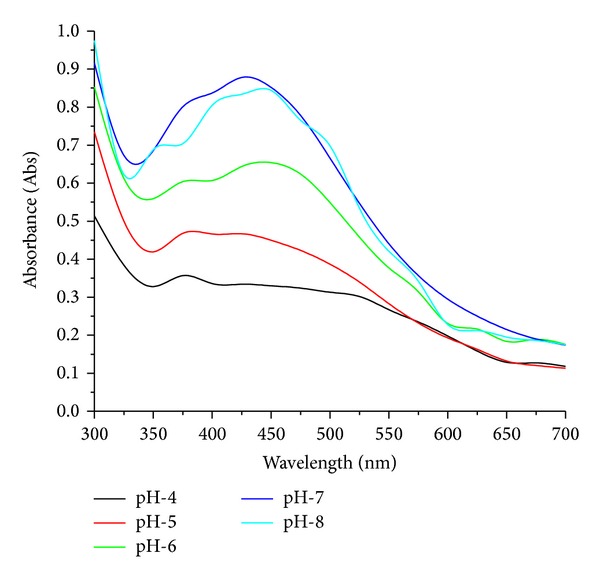
UV-visible absorption spectra of biosynthesized AgNps at different at pH.

Biosynthesis of AgNps due to the effect of varying different pH's from 4 to 8 by endophytic fungi *Penicillium *sp. is depicted in Figure 4 by UV-visible absorption spectra. The maximum peak at pH-7.0 is about 425 nm which indicates the presence of nanoparticle with a size range between 10 and 100 nm. On the contrary decrease in pH to 4.0 did not show any peak. At low pH, protein structure gets affected and the protein gets denatured and loses its activity; thus, aggregation of nanoparticles is observed [28]. It can be concluded that protein secreted by *Penicillum *sp. in the solution for the capping of AgNps is stable at pH-7.0 but not at acidic pH which can be attributed to the stability of capping proteins. Our results correlate with Banu et al. [25, 28], which shows maximum absorbance peak at 422 nm at pH-7.0 and with Jain et al. [29], using *A. flavus* at pH-7.0.

#### 3.3.3. Effect of Temperature

**Figure 5:**
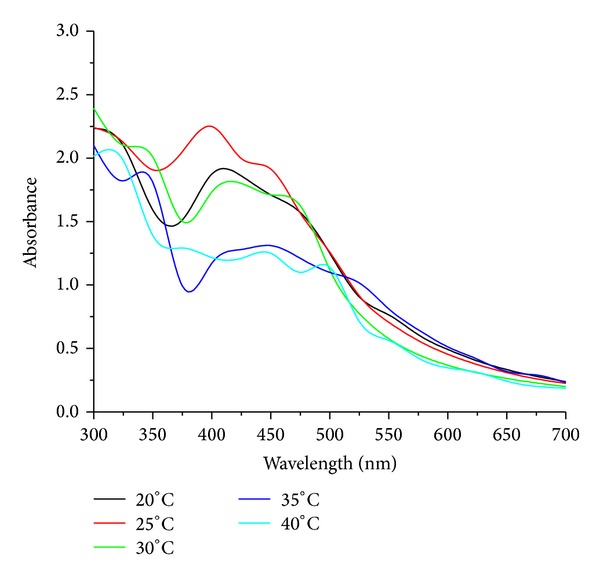
UV-visible absorption spectra of biosynthesized AgNps at different temperatures.

Temperature is an essential factor affecting AgNps production. The effect of varying temperatures on AgNps production by endophytic fungi, *Penicillium *sp., is carried out at different temperatures from 25°C to 45°C with a difference of 5°C to know the phenomenon of silver ion reduction. The maximum production of AgNps was attended at 25°C by change in color within 12–14 h quicker when compared with other temperatures and also detected by UV-visible absorption spectra the maximum peak was at 390 nm which indicates the production of AgNps [16, 29]. On the other side at high temperature of 40°C, the enzyme activity gets low so the synthesis slows down for the AgNps in the reaction Figure 5.

#### 3.3.4. Effect of Biomass Concentration

**Figure 6:**
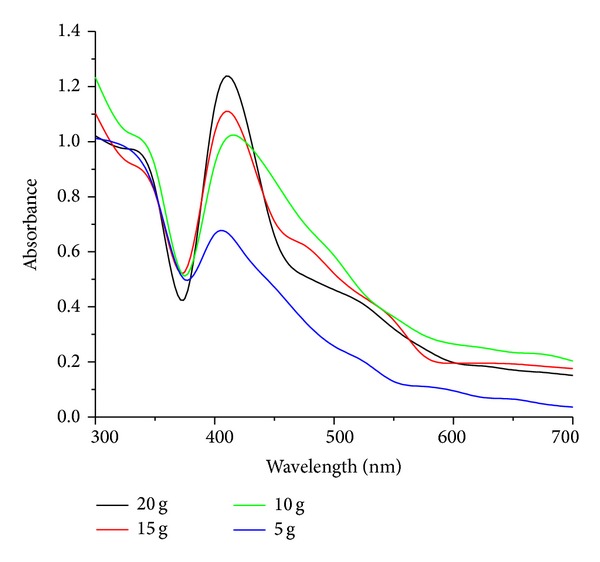
UV-visible absorption spectra of AgNps at different biomass concentrations.

Extracellular synthesis of AgNps was carried out by exposing 5 g to 20 g of wet biomass with the difference of 5 g of endophytic fungi *Penicillium *sp. in 1 mM of aqueous solution of AgNO_3_. UV-visible absorption spectra represent 15 and 20 g of wet biomass shows a maximum peak at 410 nm with no broadening or red shift of absorbance; the particles were well separated without any agglomeration, due the availability of enzymes which is sufficient for the production of nanosilver particles at pH-7 and temperature 25°C Figure 6. Our results correlate with the reports of Sunkar and Nachiyar [30] wherein they used endophytic fungi isolated by healthy leaf of *Garcinia xanthochymus* and *Aravae lanata* for the synthesis of AgNps.

### 3.4. Characterization Studies of Silver Nanoparticles

#### 3.4.1. UV-Visible Spectroscopy

**Figure 7:**
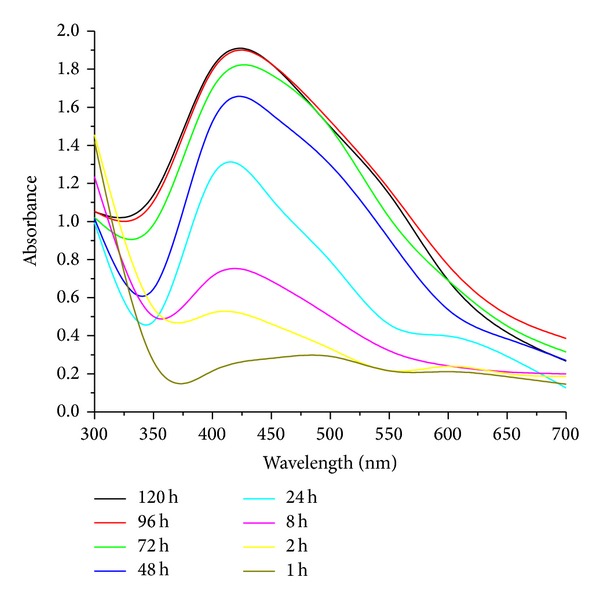
UV-visible absorption spectra of biosynthesized AgNps at different time intervals.

The extracellular synthesis of AgNps using endophytic fungi *Penicillium *sp. involves the bioreduction of silver ions in the filtrate. Reaction solution was monitored by periodic sampling of the reaction mixture at regular time intervals by using UV-visible spectroscopy. Synthesized AgNps showed maximum absorbance peak at 420 nm Figure 7. The AgNps formed were highly stable up to 120 h after the reaction. The AgNps were characterized and confirmed by TEM analysis. Similar results were observed by Ninganagouda et al., [24] who revealed plasma resonance of AgNps between 380 and 450 nm, and Sunkar and Nachiyar, who [31] revealed absorption peak at 400 nm and 423 nm by endophytic fungi isolated from leaf samples of *Garcinia Xanthochymus* and *Aravae lanata*.

#### 3.4.2. Transmission Electron Microscopy (TEM)

**Figure 8:**
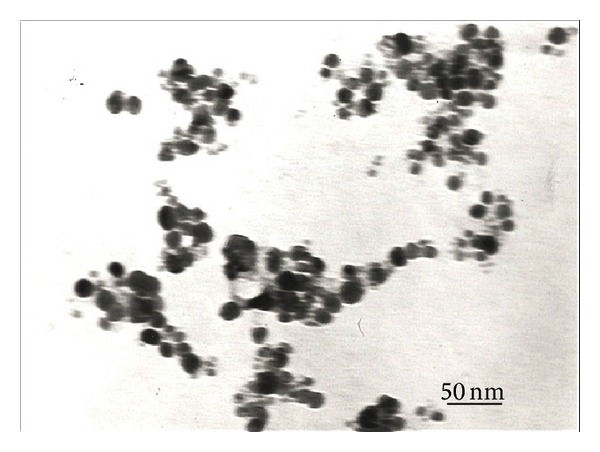
TEM image shows biosynthesized AgNps by endophytic fungi, *Penicillium *sp.

TEM measurements were carried out to determine the morphology and shape of AgNps. TEM micrograph Figure 8 revealed that the particle is spherical and well dispersed without agglomeration. The particle size of AgNps synthesized by endophytic fungi *Penicillium *sp. ranges from 25 to 30 nm. Various reports have provided evidence of extracellular synthesis of AgNps by TEM images. Ganachari et al. [32] reported well-distributed spherical shaped AgNps in the range of 5–30 nm by *Penicillium diversum *and Raheman et al. [33] also revealed spherical and polydispersive AgNps ranging from 10 to 40 nm by endophytic fungus *Pestalotia sp*. isolated from leaves of *Syzygium cumini*. (Banu et al. [25] revealed spherical shaped AgNps, with the size ranging between 3 and 20 nm by *Rhizopus stolonifer*.

#### 3.4.3. Fourier Transform Infrared Spectroscopy (FTIR)

**Figure 9:**
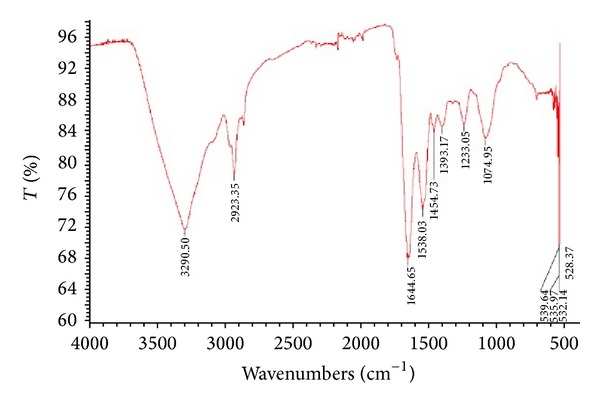
FTIR spectrum showing the presence of proteins as capping agents for AgNps synthesized by endophytic fungi, *Penicillium *sp. [20].

FTIR measurement of the dried and powdered sample was carried out to identify the possible interaction between silver and bioactive molecules, which may be responsible for synthesis and stabilization of AgNps. FTIR spectrum revealed the presence of eight bands at 1074, 1233, 1393, 1454, 1538, 1644, 2933, and 3290 cm^−1^ Figure 9. The bands at 1644, 1538, and 3290 cm^−1^ correspond to the binding vibrations of amide I and amide II band of protein, respectively, with N–H stretching's, while the 2923 cm^−1^ represents C–H stretching vibration. The bands observed at 1393, 1233, and 1074 cm^−1^ can be assigned to C–N stretching vibrations of aromatic and aliphatic amines, respectively. The observation revealed that the protein molecules not only act as reducing agent but also can act as stabilizing agent by binding to AgNps through free amino groups or cysteine residues or through electrostatic attraction of negatively charged carboxylate groups in extracellular enzyme filtrate from fungal mycelia [34].

### 3.5. Analysis of Antibacterial Activity of AgNps against MDR *E. coli* and *S. aureus* Strains

#### 3.5.1. Antimicrobial Susceptibility Test

**Figure 10:**
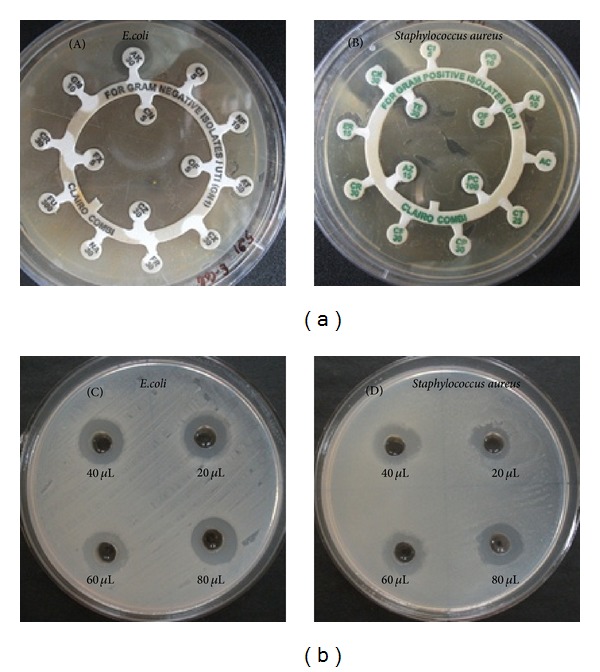
(A) Antibiotic susceptibility test of (a) *E. coli *and (b) *S. aureus. *(B)* Antibacterial activity of AgNps synthesized by endophytic fungus Penicillium* sp. against above confirmed MDR strains of (c) *E. coli *and (d) *S. aureus. *

The antibiotic susceptibility test was performed by using different antibiotic discs for both *E. coli* and *S. aureus*. For *E. coli* strain antibiotic discs such as Amikacin (AK), Norfloxacin (NF), Pefloxacin (PF), Ciprofloxacin (CI), Cefuroxime Sodium (CR), Ofloxacin (OF), Nalidixic Acid (NA), Gentamicin (GM), Cefotaxime (CX), Ceftazidime (CZ), Ceftriaxone (FR), Cefixime (FX), Cefdinir (CN), and Nitrofurantoin (AT) were used, wherein only Amikacin (AK) showed sensitivity with zone of 15 mm and resistance for other antibiotics Figure 10(a). Discs of Ciprofloxacin (CI), Cefuroxime Sodium (CR), Ofloxacin (OF), Penicillin-G (PG), Amoxicillin (AX), Amoxicillin + Clavulanic (AC), Cotrimoxazole (CT), Cephalexin (CP), Cefazolin (CF), Erythromycin (ER), Chloramphenicol (CK), Piperacillin (PC), Azithromycin (AZ), and Tetracycline (TE) were used for *S. aureus*; surprisingly only Chloramphenicol (CK) showed sensitivity against *S. aureus* with zone of inhibition of 18 mm Figure 10(b).

#### 3.5.2. Antibacterial Assay of Silver Nanoparticle against MDR *E. coli* and *S. aureus*

Antibacterial assay of biosynthesized AgNps was studied against MDR pathogenic strains (clinical isolates) of both *E. coli* and *S. aureus* using agar well diffusion method and zone of inhibition was depicted in Figure 10(B) and Table 1). AgNps solution was loaded into the wells with different concentrations of 20 *μ*L, 40 *μ*L, 60 *μ*L, and 80 *μ*L, respectively, against *E. coli* and *S. aureus. *The results revealed that AgNps were most effective against MDR *E. coli*. It was 17 mm at 80 *μ*L concentration. For MDR *S. aureus, *however, AgNps showed a mild growth inhibitory effect of 16 mm even at high concentration of 80 *μ*L when compared to MDR *E. coli *strain. Similar effects on *E. coli *and *S. aureus *pathogenic strains were observed by Kim et al., [35] and also Ninganagouda et al. [24] reported good antibacterial activity against *E. coli* using AgNps synthesized by *Aspergillus flavus*. Due to the indiscriminate use of antibiotics microorganisms have developed resistance against many antibiotics, and thus MDR strains have cropped up. A good alternative source which is ecofriendly and cost effective is only through AgNps because of their inhibitory and bactericidal effects.

## 4. Conclusion

Development of resistance to human pathogens is a challenge in field of pharmaceuticals and biomedicine. Antibiotic resistance profiles lead to fear about the emergence and reemergence of MDR pathogens. Development or modification in antimicrobial compounds to improve bactericidal potential is an area of priority in this modern era. Nanotechnology provides a good platform to modify and develop nanostructures having promising applications in various fields. Therefore, an endophytic fungus, *Penicillium* sp., isolated from healthy leaves of *Curcuma longa* (turmeric) was found to be a good producer of AgNps which remained untouched for nanoparticles production apart from being rich sources of secondary metabolites. These AgNps were proved to be powerful weapons against the MDR *E. coil* and *S. aureus *in a facial way.

## Figures and Tables

**Table 1:** Zone of inhibition of AgNps produced by the endophytic fungi *Penicillium *sp. against MDR *E. coli* and *S. aureus*.

S. no	MDR bacterial strains	Zone of inhibition (mm)			
		20 µL	40 µL	60 µL	80 µL
1	*Escherichia coli *	14 mm	15 mm	16 mm	17 mm
2	*Staphylococcus aureus *	12 mm	15 mm	15 mm	16 mm
